# An *In Vitro* Pipeline for Screening and Selection of Citrus-Associated Microbiota with Potential Anti-“*Candidatus* Liberibacter asiaticus” Properties

**DOI:** 10.1128/AEM.02883-19

**Published:** 2020-04-01

**Authors:** Alex Blacutt, Nichole Ginnan, Tyler Dang, Sohrab Bodaghi, Georgios Vidalakis, Paul Ruegger, Beth Peacock, Polrit Viravathana, Flavia Campos Vieira, Christopher Drozd, Barbara Jablonska, James Borneman, Greg McCollum, Jennifer Cordoza, Jeremiah Meloch, Victoria Berry, Lia Lozano Salazar, Katherine N. Maloney, Philippe E. Rolshausen, M. Caroline Roper

**Affiliations:** a Department of Microbiology and Plant Pathology, University of California, Riverside, Riverside, California, USA; b U.S. Department of Agriculture, Agricultural Research Service, Fort Pierce, Florida, USA; c Point Loma Nazarene University, San Diego, California, USA; d Department of Botany and Plant Sciences, University of California, Riverside, Riverside, California, USA; Chinese Academy of Sciences

**Keywords:** biocontrol, bioinoculant, natural products

## Abstract

Huanglongbing (HLB) is a destructive citrus disease that is lethal to all commercial citrus plants, making it the most serious citrus disease and one of the most serious plant diseases. Because of the severity of HLB and the paucity of effective control measures, we structured this study to encompass the entirety of the citrus microbiome and the chemistries associated with that microbial community. We describe the spatial niche diversity of bacteria and fungi associated with citrus roots, stems, and leaves using traditional microbial culturing integrated with culture-independent methods. Using the culturable sector of the citrus microbiome, we created a microbial repository using a high-throughput bulk culturing and microbial identification pipeline. We integrated an *in vitro* agar diffusion inhibition bioassay into our culturing pipeline that queried the repository for antimicrobial activity against Liberibacter crescens, a culturable surrogate for the nonculturable “*Candidatus* Liberibacter asiaticus” bacterium associated with HLB. We identified microbes with robust inhibitory activity against *L. crescens* that include the fungi Cladosporium cladosporioides and Epicoccum nigrum and bacterial species of Pantoea, Bacillus, and Curtobacterium. Purified bioactive natural products with anti-“*Ca. Liberibacter asiaticus*” activity were identified from the fungus *C. cladosporioides*. Bioassay-guided fractionation of an organic extract of *C. cladosporioides* yielded the natural products cladosporols A, C, and D as the active agents against *L. crescens*. This work serves as a foundation for unraveling the complex chemistries associated with the citrus microbiome to begin to understand the functional roles of members of the microbiome, with the long-term goal of developing anti-“*Ca*. Liberibacter asiaticus” bioinoculants that thrive in the citrus holosystem.

**IMPORTANCE** Globally, citrus is threatened by huanglongbing (HLB), and the lack of effective control measures is a major concern of farmers, markets, and consumers. There is compelling evidence that plant health is a function of the activities of the plant's associated microbiome. Using Liberibacter crescens, a culturable surrogate for the unculturable HLB-associated bacterium “*Candidatus* Liberibacter asiaticus,” we tested the hypothesis that members of the citrus microbiome produce potential anti-“*Ca*. Liberibacter asiaticus” natural products with potential anti-“*Ca*. Liberibacter asiaticus” activity. A subset of isolates obtained from the microbiome inhibited *L. crescens* growth in an agar diffusion inhibition assay. Further fractionation experiments linked the inhibitory activity of the fungus Cladosporium cladosporioides to the fungus-produced natural products cladosporols A, C, and D, demonstrating dose-dependent antagonism to *L. crescens*.

## Abstract (precis)

Globally, citrus is threatened by huanglongbing (HLB), and the lack of effective control measures is a major concern of farmers, markets, and consumers. There is compelling evidence that plant health is a function of the activities of the plant's associated microbiome. Using Liberibacter crescens, a culturable surrogate for the unculturable HLB-associated bacterium “*Candidatus* Liberibacter asiaticus,” we tested the hypothesis that members of the citrus microbiome produce potential anti-“*Ca*. Liberibacter asiaticus” natural products with potential anti-“*Ca*. Liberibacter asiaticus” activity. A subset of isolates obtained from the microbiome inhibited *L. crescens* growth in an agar diffusion inhibition assay. Further fractionation experiments linked the inhibitory activity of the fungus Cladosporium cladosporioides to the fungus-produced natural products cladosporols A, C, and D, demonstrating dose-dependent antagonism to *L. crescens*.

## INTRODUCTION

Huanglongbing (HLB) is a serious disease of citrus and the major threat to citriculture worldwide. In the United States, HLB is associated with a Gram-negative, phloem-limited alphaproteobacterium, “*Candidatus* Liberibacter asiaticus,” with several different strains of “*Ca*. Liberibacter asiaticus” reported in association with citrus (1–3). This bacterium is spread by insect psyllid vectors; the psyllid vector in the United States is the Asian citrus psyllid (ACP) Diaphorina citri. Both the vector and the bacterium are invasive species in the United States. Symptoms of the disease include leaf chlorosis, blotchy mottle, limb dieback, root loss, phloem plugging, and overall sieve element collapse (4, 5). Diseased trees produce small, bitter, hard, unevenly colored, and misshapen fruit. These fruits are unmarketable for juicing because the disease results in acidic, salty, and off-flavor juice. In addition to the unpalatable flavor, fruit borne of trees with severe HLB symptoms exhibit severe morphological distortions and seed discoloration, rendering them unsuitable for fresh-market sale (6, 7). Infected trees decline rapidly and die within a few years of becoming infected, and HLB can spread throughout an orchard in a short period of time, especially when environmental conditions are favorable or mitigation measures are not applied (8). All commercial citrus varieties are susceptible to HLB (9, 10). Current management of HLB relies heavily on vector control via insecticide applications, and the development of alternative effective management strategies is ongoing (11, 12). Section 18 emergency registration was approved in Florida for the use of the antibiotics streptomycin sulfate and oxytetracycline hydrochloride in citrus, and the studies regarding the efficacy of these antibiotics against HLB are ongoing (11–13), with a recent study indicating that spray applications of oxytetracycline are ineffective at mitigating HLB (14).

A diverse community of microorganisms is associated with plants, collectively referred to as a plant’s microbiome, and includes the collection of microbes associated with the rhizosphere (the soil-root interface), the phyllosphere (epiphytic, aerial surfaces), and the endosphere (internal tissues) (15). Spatial and environmental factors as well as host immunity and microbe-microbe interactions can shape the microbiome community structure in these plant compartments (16–19). Moreover, under disease conditions, microbial pathogens directly or indirectly interact with the host microbiome as well as the host itself. Because of the HLB epidemic and the lack of long-term sustainable effective control measures, there is an increased focus on the citrus microbiome and how it relates to the HLB disease phenotype that encompasses the entirety of the citrus microbial community and its associated chemistries (20–22). High-throughput sequencing (HTS) technologies have significantly increased our knowledge regarding the members of plant-associated microbiomes, including those of citrus. However, besides pathogens and some well-studied symbionts, the vast majority of the functions of the plant microbiome are unknown, colloquially referred to as microbial “dark matter” (23). Their intimate host associations suggest that these microbes may possess enormous untapped potential for promoting plant health, but the inherent complexity of these communities and their associated chemistries complicate efforts to decipher their respective contributions (24, 25).

The next frontier in microbiome research is to move beyond microbial community profiling to define specific microbial contributions to phenotypes, such as plant health and disease outcomes (26). These efforts are expedited by coupling big data sets derived from HTS technologies with reductionist experiments using microbial isolates in singlet or consortia that are derived from a given microbiome. Thus, establishing and maintaining culture collections alongside cognate culture-independent HTS data sets is a key component of unraveling the complexity of microbial functions within a host’s microbiome. HTS technologies in plant microbiomes have also enabled the field of microbial biocontrol to shift from single-agent control studies toward holistic, community-based investigations on the comprehensive microbiome of a given system (27). However, the market for biocontrol agents or microbially derived natural product-based disease control applications is still heavily rooted in culture-dependent studies, because the development of microbe-derived formulations for commercial purposes requires culturable isolates that can be broadened to scaled-up fermentations. Thus, the integration of culture collections with culture-independent microbiome data sets is particularly relevant to the field of biocontrol and natural product-based disease control research.

Enduring biological control requires microbes that are adapted to changing host disease states as part of an integrated management strategy. The most successful biocontrol agents are those tailored to their target environment and that are capable of thriving across healthy and diseased host states (28). Rhizobium rhizogenes K84 (29) is a model integrated biocontrol agent and used, along with the derived strain Rhizobium rhizogenes K1026 (30), to combat infection of Agrobacterium tumefaciens in the rhizosphere of susceptible plants (31). This biocontrol agent was isolated from A. tumefaciens-infested rhizospheres where these two microbes evolved to compete with one another through an elegant interaction mechanism mediated by the antibiotic agrocin 84, allowing *R. rhizogenes* to specifically inhibit virulent A. tumefaciens strains carrying specific Ti plasmids (32). A seemingly logical starting point for biocontrol bioprospecting efforts from within a host’s microbiome would focus on healthy or asymptomatic hosts. However, utilizing the success of *R. rhizogenes* K84 and K1026 as a paradigm for the development of an effective biocontrol agent, it has been proposed that bioprospecting for biocontrol candidates should also include the microbiota from symptomatic hosts (33, 34). A study in tomato also indicated that a pathogen-prevalent environment was a good source for isolating biocontrol agents for the vascular bacterial pathogen of solanaceous plants, Ralstonia solanacearum (35). These conditions select for candidate biocontrol agents capable of sustaining themselves within the parameters of the diseased plant environment. Moreover, these microorganisms interface with the pathogen either directly or indirectly and are potentially under selective pressure to engage in competitive interactions with the pathogen.

The collective aims of this work were to map the spatial anatomy of the citrus microbiome in different tissue niches of the tree (leaves, stems, and roots) and to mine those same niches for culturable microbiota to build a repository of citrus-associated microorganisms that dwell in the HLB disease environment and screen this repository for potential anti-“*Ca*. Liberibacter asiaticus” bioinoculants. To accomplish this, we utilized a high-throughput culturing and taxonomic identification pipeline that allows for the rapid identification of large cohorts of culturable microbiota based on bulk-culturing techniques augmented with amplicon-based HTS technologies that alleviated the initial need for laborious subculturing into pure culture. We then isolated a subset of these microbial cohorts into pure culture to create a repository of axenic citrus microbial isolates. Operating under the premise that members of the citrus microbiome could be developed into HLB suppressors, we tested the hypothesis that members of the citrus microbiota can compete with “*Candidatus* Liberibacter asiaticus” through antibiosis. Efforts to culture the “*Candidatus* Liberibacter asiaticus” bacterium are ongoing and remain a large focus of the research community working on the HLB pathosystem (36). However, the bacterium remains unculturable. Thus, “*Ca*. Liberibacter asiaticus” is not amenable to manipulation *in vitro*, which poses severe limitations on developing bioassays to screen compounds that target “*Ca*. Liberibacter asiaticus” directly. Because of this, we turned to *L. crescens*, the only cultivable species belonging to the *Liberibacter* genus (37). *L. crescens* has also been detected in citrus, and several studies have established it as a suitable *in vitro* model organism for “*Ca*. Liberibacter asiaticus” (38, 39). We integrated a robust *in vitro* agar diffusion inhibition bioassay into our culturable microbiome pipeline that utilizes *L. crescens* as a target to identify citrus-associated bacteria and fungi that produce metabolites that inhibit its growth. This *in vitro* screening pipeline was validated by isolating natural products cladosporols A, C, and D with antimicrobial activity from the *L. crescens*-antagonistic fungus *C. cladosporioides*, thereby providing foundational data for the development of native citrus microbiome-derived therapeutic methods with potential application in HLB management practices and possibly other plant pathosystems as well.

## RESULTS

### Accessing the culturable citrus microbiome using a high-throughput bulk-culturing pipeline.

**FIG 1:**
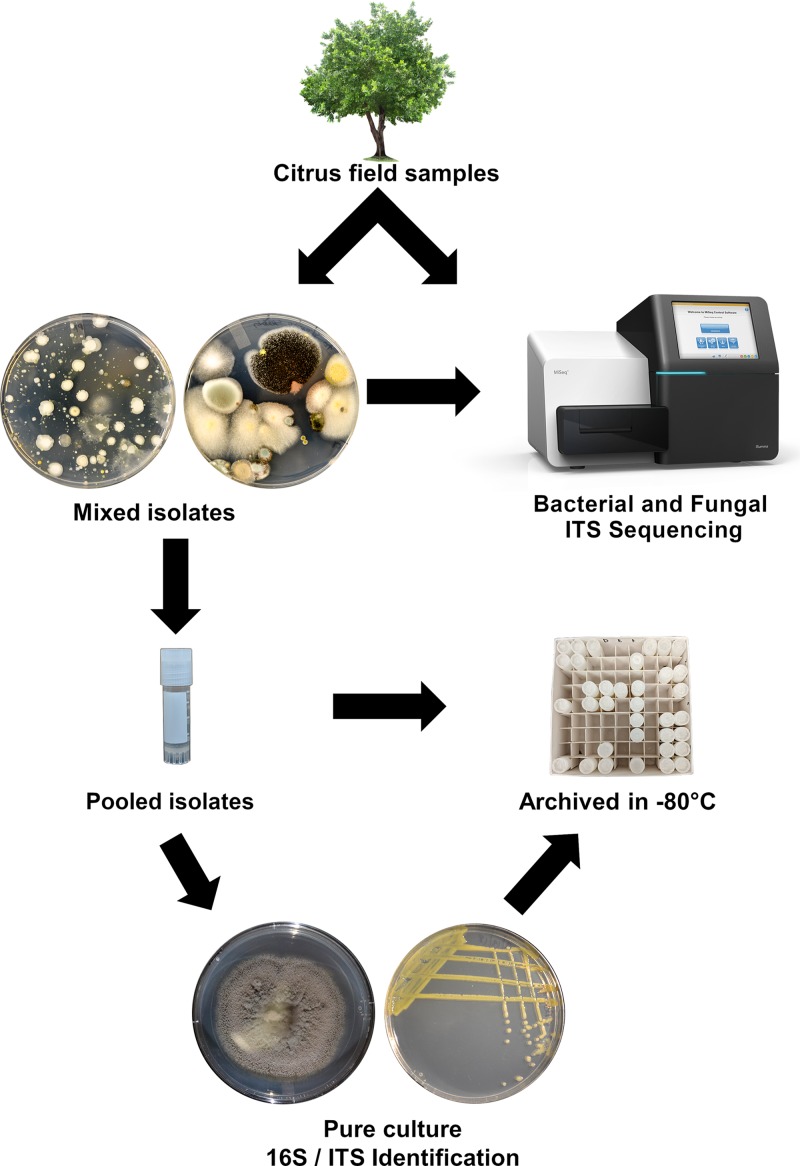
High-throughput bulk-culturing pipeline for construction of the citrus-cultured microbiome repository. Fungi and bacteria were cultured from citrus leaves, stems, and roots onto TSA and PDA medium at 28°C for 4 days. Bulk cultures were harvested from the plates, archived as a mixture in 25% glycerol, and stored at –80°C in cryovials. Aliquots of the archived microbial mixtures were assessed via ITS sequencing to determine the diversity captured through culturing. Microbial diversity was also assessed using culture-independent methods from the same citrus tissues that were used for the culture-dependent analyses. Individual isolates were obtained via subculturing from the mixed cultures, stored as part of the citrus microbiome repository, and screened in the bioassay against *L. crescens* BT-1.

We utilized a bulk-culturing pipeline to initially assign taxonomic classification to the microbes obtained from our culturing efforts before isolating them into pure culture (Fig. 1). Taxonomic assignment of the bulk cultures enabled us to obtain federal permits (P526P-18-01661 and P526P-17-04593) to import into California 248 bulk culture tubes that contained no known regulated citrus pathogens as determined by the amplicon-based HTS analyses of both bacteria and fungi. We then performed subculturing and isolation into pure culture in Riverside, CA (Fig. 1). Both the bulk cultures and individual isolates derived from the bulk cultures that were permitted and shipped to Riverside, CA, from Fort Pierce, FL, formed the basis of our culture repository.

### Spatial mapping of the culture-dependent and -independent citrus microbiome.

Tissues were not surface sterilized prior to the culture-independent or culture-dependent protocols, so the taxa reported here represent epiphytic and endophytic microorganisms.

### (i) Culture dependent.

**FIG 2:**
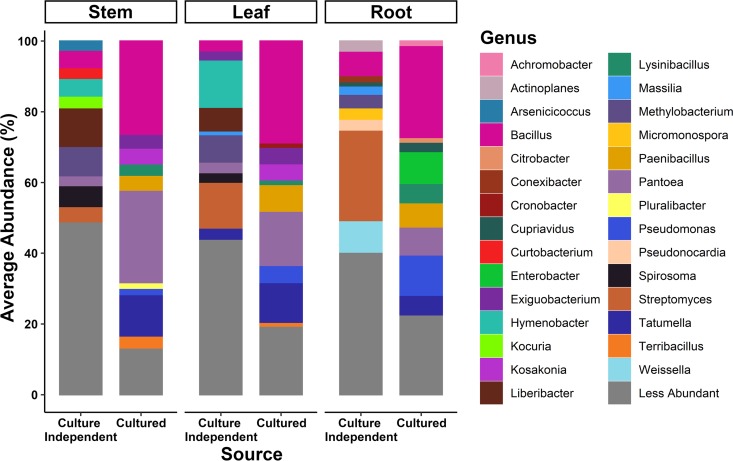
Diversity within the culture-independent and culturable fractions of the bacteriomes of citrus leaves, stems, and roots. Plots illustrate the relative abundances of the bulk-cultured bacterial genera across leaf, stem, and root tissues (culture independent) compared to their cognate cultured bacterial communities derived from the same samples (culture dependent). Colors denote different genera with the most the 29 most abundant genera labeled.

**FIG 3:**
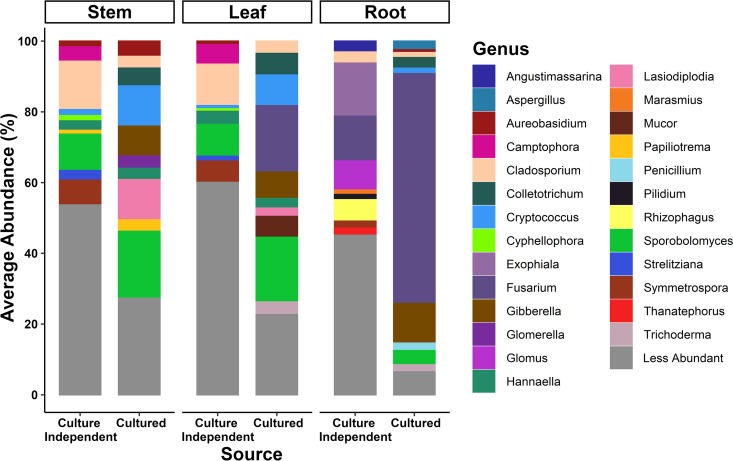
Diversity within the culture-independent and culturable fractions of the mycobiomes of citrus leaves, stems, and roots. Plots illustrate the relative abundances of the bulk-cultured fungal genera across leaf, stem, and root tissues (culture independent) compared to their cognate cultured fungal communities derived from the same samples (culture dependent). Colors denote different genera with the 29 most abundant genera labeled.

Our study utilized primers that target the bacterial intergenic spacer (ITS) region, whereas other published citrus microbiome studies generally utilized primers that target the 16S rRNA gene. Bacterial ITS primers can provide finer taxonomic resolution than do bacterial 16S rRNA gene primers and can sometimes provide species-level identification (40). After removing low-abundance operational taxonomic units (OTUs) (average abundance, <1 count across all samples) from amplicon-based HTS data of the bulk cultures, we obtained 863 OTUs in the cultured leaf bacteriome, 679 OTUs in the cultured stem bacteriome, and 880 OTUs in the cultured root bacteriome from the archived bulk-cultured samples. We obtained 467 OTUs in the cultured leaf mycobiome, 478 OTUs in the cultured stem mycobiome, and 216 OTUs in the cultured root mycobiome from the archived bulk-cultured samples (Fig. 2 and 3 and Tables 1 and 2). The 10 most abundant bacterial genera found in all three tissue types are listed in Table 1 and presented in Fig. 2. Isolates belonging to the genera Bacillus, Pantoea, Tatumella, Paenibacillus, Pseudomonas, and Lysinibacillus were obtained in bulk culture from all three tissue types. A list of all bacterial OTUs, taxa, and metadata associated with each sample can be found in Tables S1 to S3 in the supplemental material. The 10 most abundant fungal genera isolated in bulk cultures from leaves, stems, and roots in terms of relative abundance can be found in Table 2 and Fig. 3. The fungal isolates identified by HTS in these bulk cultures that were common to all three tissue types were from the genera Sporobolomyces, Cryptococcus, Fusarium/Gibberella, Colletotrichum, Cladosporium, and Aureobasidium. A list of the fungal OTUs (average abundance, >1 count across all samples), taxa, and metadata associated with each sample can be found in Tables S4 to S6.

**TABLE 1:** Relative abundance percentages of the 10 most abundant genera of the citrus bacteriome

Culture dependence	Taxon in citrus bacteriome in different tissue compartments (% relative abundance)^*a*^		
	Leaf	Stem	Roots
Dependent	***Bacillus*** (37.4)	***Bacillus*** (34.7)	***Bacillus*** (28.5)
	***Pantoea*** (12.3)	***Pantoea*** (20.4)	*Enterobacter* (11.1)
	***Tatumella*** (12)	***Tatumella*** (12.5)	***Pseudomonas*** (9.1)
	***Paenibacillus*** (8.6)	***Paenibacillus*** (5.6)	***Lysinibacillus*** (7.6)
	*Exiguobacterium* (5.2)	*Exiguobacterium* (5.1)	***Paenibacillus*** (7.1)
	*Kosakonia* (4.2)	*Terribacillus* (3.9)	***Pantoea*** (6.4)
	***Pseudomonas*** (2.5)	*Kosakonia* (3.3)	***Tatumella*** (3.8)
	***Lysinibacillus*** (1.3)	***Lysinibacillus*** (2.4)	*Cupriavidus* (2.5)
	*Brevibacillus* (1.2)	***Pseudomonas*** (1.5)	*Achromobacter* (1.0)
	*Terribacillus* (1.1)	*Psychrobacillus* (1.1)	*Citrobacter* (1.0)
Independent	*Liberibacter* (12.2)	*Liberibacter* (11.0)	***Streptomyces*** (24.4)
	***Streptomyces*** (11.8)	*Spirosoma* (8.7)	*Weissella* (15.5)
	*Armatimonadetes* (8.6)^*b*^	*Methylobacterium* (7.6)	*Flavobacteriales* (6.7)^*b*^
	*Pantoea* (5.4)	*Hymenobacter* (6.2)	*Pseudonocardia* (6.2)
	*Massilia* (5.3)	*Massilia* (5.7)	***Bacillus*** (5.8)
	*Hymenobacter* (5.0)	“*Candidatus* Walczuchella” (5.2)	*Micromonospora* (2.6)
	*Tatumella* (4.4)	***Bacillus*** (4.4)	*Cupriavidus* (1.9)
	*Methylobacterium* (3.5)	*Kocuria* (4.3)	*Mycolicibacterium* (1.9)
	*Spiroplasma* (2.7)	*Pantoea* (4.2)	*Mycoplasma* (1.7)
	***Bacillus*** (2.3)	***Streptomyces*** (4.1)	*Mycobacterium* (1.4)

a Taxa that are conserved across all three tissue types are indicated in bold.

b Taxa that could not be identified to the genus level.

**TABLE 2:** Relative abundances of the 10 most abundant genera of the citrus mycobiome

Culture dependence	Taxon in citrus mycobiome in different tissue compartments (% relative abundance)^*a*^		
	Leaf	Stem	Root
Dependent	***Sporobolomyces*** (20.8)	***Sporobolomyces*** (16.0)	*Fusarium* (50.9)
	***Cryptococcus*** (10.5)	***Cryptococcus*** (14.1)	***Gibberella*** (19.7)
	***Gibberella*** (9.4)	*Lasiodiplodia* (11.8)	***Colletotrichum*** (6.0)
	*Fusarium* (9.2)	***Gibberella*** (8.9)	*Penicillium* (3.8)
	*Mucor* (7.6)	***Colletotrichum*** (4.9)	*Aspergillus* (2.3)
	***Colletotrichum*** (6.7)	***Aureobasidium*** (4.1)	*Trichoderma* (2.3)
	***Cladosporium*** (3.9)	*Papiliotrema* (3.5)	***Cladosporium*** (1.9)
	*Trichoderma* (3.1)	*Glomerella* (3.4)	***Sporobolomyces*** (1.2)
	*Lasiodiplodia* (2.7)	***Cladosporium*** (3.4)	***Cryptococcus*** (0.95)
	***Aureobasidium*** (2.1)	*Hannaella* (3.4)	***Aureobasidium*** (0.93)
Independent	***Cladosporium*** (13.0)	***Cladosporium*** (15.2)	*Exophiala* (17.8)
	*Camptophora* (9.2)	*Camptophora* (9.24)	*Fusarium* (16.9)
	*Symmetrospora* (7.6)	*Sporobolomyces* (9.01)	*Glomus* (8.0)
	*Sporobolomyces* (6.9)	*Symmetrospora* (7.67)	Glomeromycota (6.1)^*b*^
	*Exophiala* (3.7)	*Strelitziana* (3.24)	*Rhizophagus* (4.0)
	*Uwebraunia* (3.6)	*Colletotrichum* (2.85)	*Angustimassarina* (3.5)
	*Alternaria* (2.6)	**Didymellaceae** (2.18)^*b*^	Sordariales (3.0)^*b*^
	*Fusarium* (2.2)	*Cyphellophora* (1.60)	***Cladosporium*** (2.1)
	**Didymellaceae** (2.2)^*b*^	*Hannaella* (1.57)	**Didymellaceae** (2.0)^*b*^
	*Strelitziana* (2.0)	*Aureobasidium* (1.47)	*Thanatephorus* (1.7)

a Taxa that are conserved across all three tissue types are indicated in bold.

b Taxa that could not be identified to the genus level.

### (ii) Culture independent.

The culture-independent data presented here are a subset of the large-scale citrus microbiome HTS data set that was deposited in the Sequence Read Archive of the National Center for Biotechnology Information (accession numbers SRP127690 and SRX3520308 to SRX3520607) (20). Here, we provide a detailed description of the biology underlying these HTS data in the context of the citrus tissues from which they were derived and use it as a foundation to compare to the culturable citrus microbiomes obtained from the same samples that were utilized to generate the culture-independent HTS. In brief, after removing low-abundance OTUs (<1 average abundance per sample), leaf tissues contained 5,326 bacterial OTUs, stem tissues contained 4,319 bacterial OTUs, and root tissues contained 8,681 bacterial OTUs. The 10 most abundant bacterial genera in leaf tissues in terms of relative abundance in the culture-independent data set are listed in Table 1 and Fig. 2. Of the 10 most abundant genera in the culture-independent study, Streptomyces and *Bacillus* were common to all three tissue compartments. The list of bacterial OTUs, taxa, and metadata associated with each sample and percentage of OTUs of citrus origin obtained for the culture-independent data set can be found in Tables S4 to S6. After removing low-abundance OTUs (<1 average abundance per sample), leaf tissues contained 1,638 fungal OTUs, stem tissues contained 1,593 fungal OTUs, and root tissues contained 1,663 fungal OTUs. The 10 most abundant fungal genera associated with citrus leaves in terms of relative abundance in the culture-independent data set are listed in Table 2 and presented in Fig. 3. The fungal taxa present in all three tissue compartments were the genus *Cladosporium* and the family Didymellaceae. The list of all fungal OTUs, taxa, and metadata associated with each sample and percentage of OTUs of citrus origin obtained for the culture-independent data set can be found in Tables S4 to S6.

### Representation of species richness in the cultured citrus microbiome.

**FIG 4:**
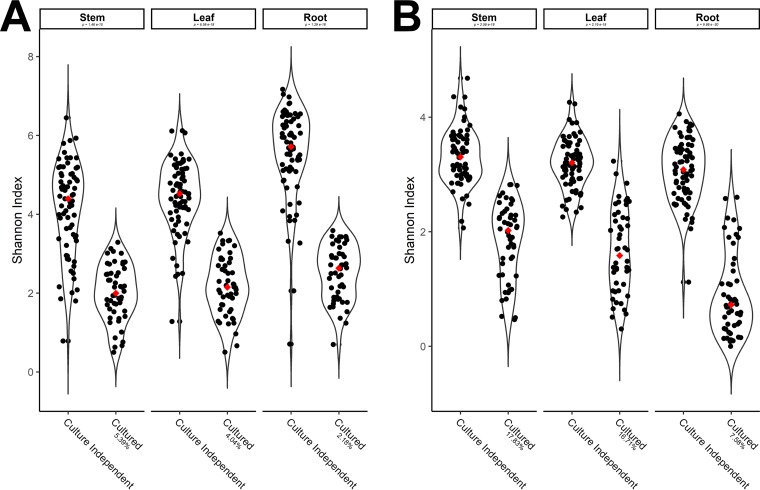
Passage through culture medium produces diversity shifts in citrus-associated microbiota. (A and B) Violin plots illustrating Shannon’s alpha-diversity index scores of the citrus bacteriome and its cultured counterparts, per tissue (A) and the citrus mycobiome and its cultured counterparts, per tissue (B). Red diamonds represent the medians of each sample group. The cultured portion of the bacteriome represents 4.0% of the culture-independent taxa in the leaves, 5.4% of the culture-independent taxa in the stems, and 2.2% of the culture-independent taxa in the roots. The cultured mycobiome represents 16.7% of the culture-independent taxa in the leaves, 17.8% of the culture-independent taxa in the stems, and 7.6% of the culture-independent taxa in the roots. *P* values indicate the significance of the difference in alpha-diversity measures between culture-independent and culture-dependent samples per tissue, obtained via a Kruskal-Wallis with *post hoc* Dunn test, using Bonferroni correction (*P < *0.05). Percent values indicate the proportions of culture-independent OTUs found in cultured microbiome samples.

Compared to the culture-independent data from the field samples from which the bulk cultures were derived, the cultured portion of the bacteriome represents 4.0% of the culture-independent taxa in the leaves, 5.4% of the culture-independent taxa in the stems, and 2.2% of the culture-independent taxa in the roots. The cultured mycobiome captured in this study represents a higher percentage of fungal taxa present in the comprehensive microbiome than what was represented for the bacterial taxa. Specifically, the cultured mycobiome represents 16.7% of the culture-independent taxa in the leaves, 17.8% of the culture-independent taxa in the stems, and 7.6% of the culture-independent taxa in the roots. These data taken together indicate that, not surprisingly, alpha diversity is significantly reduced when examining the culturable portion of the microbiome. This culture-imposed bottleneck was observed for each tissue type sampled (Fig. 4). Overall, as expected, there were significant differences (*P < *0.05, Kruskal-Wallis with *post hoc* Dunn test, using Bonferroni correction) in alpha diversity indexes between culture-dependent and -independent methods. Percent values indicate the proportions of culture-independent OTUs found in cultured microbiome samples.

### Isolation and identification of individual microbial isolates.

Considering that “*Ca*. Liberibacter asiaticus” is initially introduced by the Asian citrus psyllid into aerial citrus tissues via feeding on new vegetative leaf growth (flush), we focused our subculturing to pure culture efforts on the aerial tissues of citrus (leaves and stems) to screen for potential anti-“*Ca*. Liberibacter asiaticus” bioinoculants. Overall, we obtained 1,326 pure culture isolates from a subset (28 tubes) of the 148 bulk-culture tubes that were derived from the leaf and stem tissues. Of these, 49.17% (652 isolates) were identified to the genus level to be bacteria, and 7.39% (98 isolates) were identified to the genus level to be fungi. The remaining 43.44% were either recalcitrant to identification or have not yet been identified to the genus level. The pure isolates were representative of the colony morphotypes observed on the mixed-culture plates. The 10 most abundant bacterial genera isolated into pure cultures from the leaf and stem tissues are *Bacillus*, *Pantoea*, *Curtobacterium*, Rosenbergiella, Microbacterium, *Pseudomonas*, Kosakonia, Lysinibacillus, Paenibacillus, and Erwinia. Other bacterial genera represented in the culture repository can be found in Fig. S1. All of these taxa were identified by 97% homology to 16S rRNA gene nucleotide sequences from specimens posted in the Ribosomal Database Project (41) or the NCBI database. The 10 most abundant fungal genera isolated into pure culture from leaves and stems are Mucor, *Cryptococcus*, *Aureobasidium*, *Cladosporium*, *Fusarium*, Penicillium, Coniochaeta, Papiliotrema, *Colletotrichum*, and Alternaria. Other fungal genera represented in the culture repository can be found in Fig. S2. All of these taxa were identified by 97% homology to ITS rRNA gene nucleotide sequences from specimens posted in the NCBI database (42).

### Identification of *L. crescens*-inhibitory microbes.

**FIG 5:**
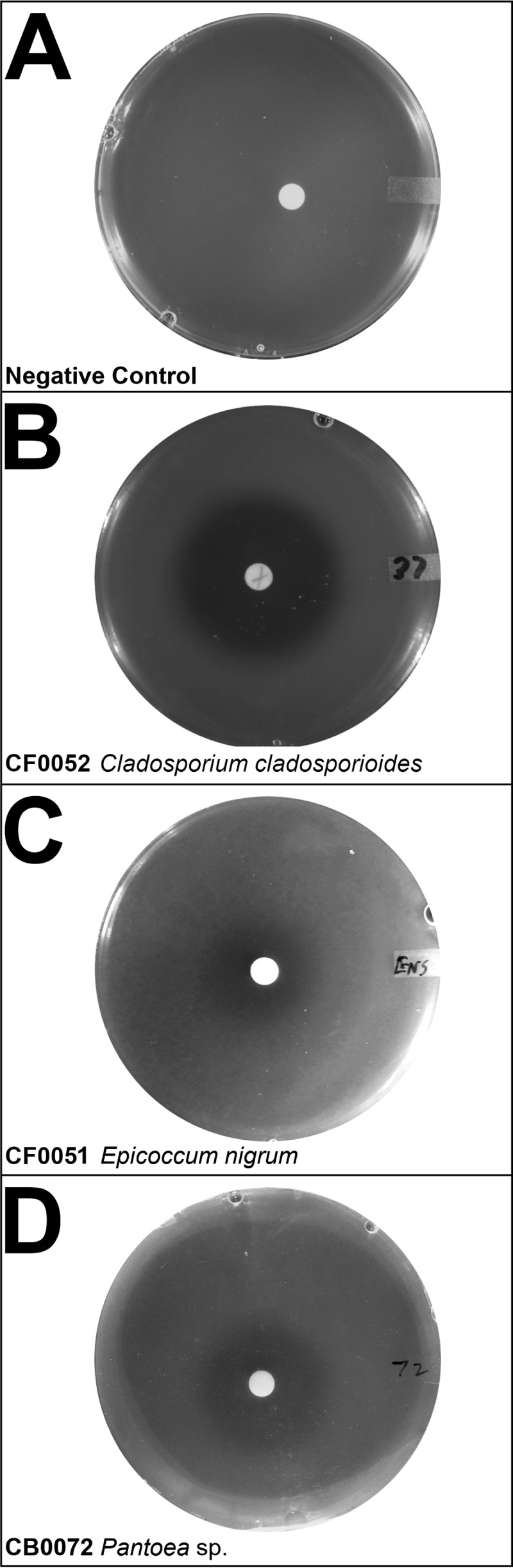
Liberibacter crescens agar diffusion inhibition assay. (A to D) Images of assay plates from the *in vitro* diffusion assay, showing uninhibited *L. crescens* BT-1 growth on a negative-control plate (A), a halo of *L. crescens* BT-1 growth inhibition around a disk containing supernatant from *C. cladosporioides* (CF0052) (B), a halo of *L. crescens* BT-1 growth inhibition around a disk containing supernatant from *E. nigrum* (CB0051) (C), and a halo of *L. crescens* BT-1 growth inhibition around a disk containing supernatant from *Pantoea* sp. isolate CB0072 (D). Fifty microliters of MeOH was applied and evaporated off the disk prior to placement on the top agar.

To screen our microbial library for competitive interactions with “*Ca*. Liberibacter asiaticus,” we utilized the culturable close relative *L. crescens* as a functional proxy for screening for microbial antagonists. We initially adapted a solid agar-based bioassay using a dilution series of spectinomycin, an antibiotic known to inhibit the growth of *L. crescens* (Table S7). Once this assay was established, we then initiated testing crude supernatants obtained from approximately 17% (244 isolates) of the pure cultures in a medium-throughput format. We identified *L. crescens*-inhibitory bacterial and fungal isolates via the presence of zones of growth inhibition around the disks loaded with their respective supernatants, indicating the presence of antimicrobial compounds (Fig. 5). These included three fungi belonging to the genera *Cladosporium* and *Epicoccum* and nine bacteria belonging to the genera *Pantoea*, *Bacillus*, and *Curtobacterium* (Table 3).

**TABLE 3:** Crude supernatants from citrus-associated microbes that are inhibitory to Liberibacter crescens BT-1 in agar diffusion bioassays

Isolate	Tissue origin	Identification	Zone of inhibition diam (cm)^*a*^
CF0052	Leaf	Cladosporium cladosporioides	3.40 ± 0.32
CF0053	Leaf	*C. cladosporioides*	2.58 ± 0.38
CF0051	Stem	*Epicoccum nigrum*	1.58 ± 0.16
CB0072	Leaf	*Pantoea* sp.	2.65 ± 0.28
CB00729	Leaf	*Bacillus* sp.	2.6
CB00687	Leaf	*Bacillus* sp.	3.33 ± 0.39
CB00912	Leaf	*Bacillus* sp.	3.8
CB00904	Leaf	*Bacillus* sp.	1.8
CB00892	Leaf	*Curtobacterium* sp.	1.35
CB00909	Leaf	*Bacillus* sp.	1.78
CB00893	Stem	*Bacillus* sp.	1.00
CB00945	Leaf	*Curtobacterium* sp.	1.00

a The negative control had MeOH only and had a zone of inhibition diameter of 0.00 ± 0.00 cm.

### Bioassay-guided isolation of cladosporols Cladosporium cladosporioides.

**FIG 6:**
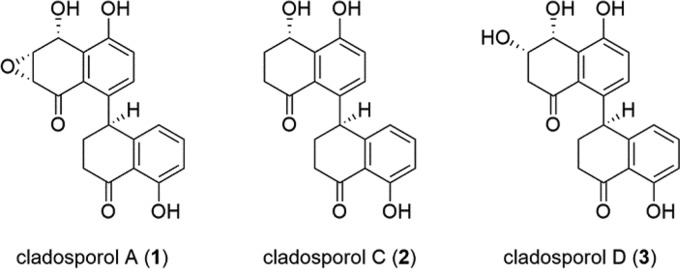
Structures of cladosporols A (compound 1), C (compound 2), and D (compound 3).

**FIG 7:**
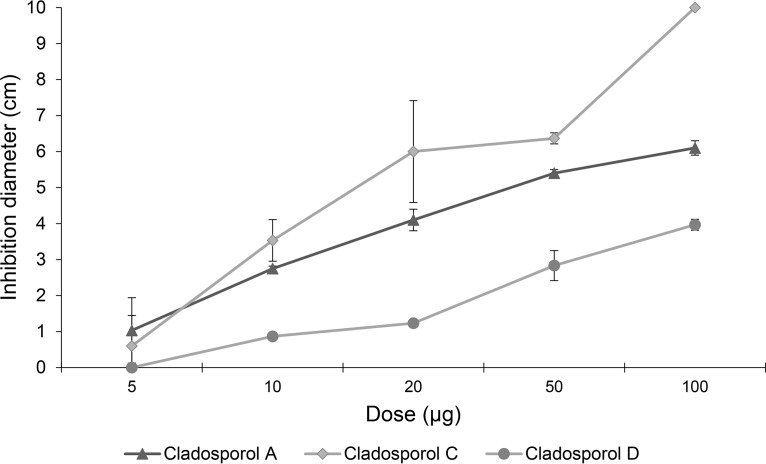
Dose-response assay for cladosporols. Cladosporols A, C, and D display dose-dependent activity in the *L. crescens* inhibition assay.

As a proof of concept, we focused our natural product isolation efforts on the fungal strain exhibiting the largest and most robust inhibition, *C. cladosporioides* CF0052. This strain was propagated in potato dextrose broth (PDB), the organic-soluble metabolites were extracted with ethyl acetate and fractionated using flash column chromatography and high-performance liquid chromatography (HPLC), and active fractions were identified using the *L. crescens* inhibition assay. Flash column fractions 3 to 5 all strongly inhibited *L. crescens* growth (inhibition diameters, 6.0 cm, 6.4 cm, and 5.7 cm, respectively). The fractions were subjected to HPLC to give three pure compounds. The ^1^H nuclear magnetic resonance (NMR) spectra of these compounds each contained a highly deshielded singlet (11.5 to 12.5 ppm) consistent with strongly hydrogen-bonded phenols. A search of the AntiMarin natural products database (43) for *Cladosporium* metabolites with phenols capable of such hydrogen bonding yielded 29 compounds; of these, cladosporols A (compound 1, formula C_20_H_16_O_6_), C (compound 2, formula C_20_H_18_O_5_), and D (compound 3, formula C_20_H_18_O_6_) (44, 45) had molecular masses consistent with those observed by liquid chromatography-mass spectrometry (LC-MS) (*m/z* 351.05 [M-H]^−^, 337.08 [M-H]^−^, and 353.07 [M-H]^−^, respectively) (Fig. 6). A comparison of the ^1^H and ^13^C NMR spectra for each compound with the literature spectra for the cladosporols confirmed the identities of compounds 1 to 3 as the major bioactive compounds from *C. cladosporioides*. The NMR and LC-MS data for fractions 4 and 5 suggested that these also contained compounds 1 and 2, along with other yet-to-be-identified metabolites. Furthermore, purified compounds 1 to 3 from *C. cladosporioides* showed dose-dependent inhibition of *L. crescens* in the *in vitro* disk diffusion inhibition assay (Fig. 7). All showed comparable dose-response curves, with compound 2 showing slightly higher inhibition at each concentration tested.

## DISCUSSION

Specific members or consortia of plant microbiomes can provide protection against plant pathogens through a variety of mechanisms ranging from niche displacement, production of antimicrobial compounds, and activation of induced systemic resistance (46–52). Reductionist experiments facilitate mechanistic studies to elucidate the underlying biology of a system, rendering culture collections an important translational research tool to bridge big HTS data sets with biologically relevant activities. These resources enable critical inquiries into specific microbial interactions, such as linking functional phenotypes like pathogen suppression to specific microbiome constituents and their respective bioactive chemistries (53). For this study, we designed a pipeline that allowed us to assign taxonomy to bulk cultures obtained from citrus tissues. This conveniently expedited taxonomic assignments by initially bypassing the need to isolate into pure culture. Moreover, our methodology was derived out of necessity to adapt to the regulatory logistics of working with “*Ca*. Liberibacter asiaticus”-infected citrus tissues in California. HLB has only recently been confirmed in California (2012), and prior to that, the state was considered to be HLB free (54). “*Ca*. Liberibacter asiaticus” is a quarantine pathogen for the state of California, and as such, scientists in California are not permitted to import citrus tissues containing live “*Ca*. Liberibacter asiaticus.” Tissue sampling and bulk culturing were performed in Florida, where bulk cultures were archived and stored temporarily. In California, HTS libraries were constructed, sequenced, and analyzed using DNA isolated from the bulk cultures. Once taxonomy was assigned to the microorganisms archived in Florida and confirmed to contain no known pathogens of citrus, federal importation permits were obtained, and the bulk cultures were imported to California, where isolation to pure culture was initiated. Inherent to any culturing process, the standardized growth media and conditions utilized in this study imposed a bottleneck on isolates derived from citrus tissue and significantly reduced bacterial and fungal species richness compared to that in the *in planta* microbiome. Regardless, the citrus culture repository successfully captured many high-abundance bacterial and fungal taxa that were identified in the culture-independent data set, representing a higher-than-expected proportion of taxa observed *in planta* across tissue types. Our citrus microbial collection includes metadata and barcode sequence for each microbial isolate (20), and as we develop the repository, we expand our collection sites to include other geographic regions, such as California, where HLB is just beginning to manifest, along with a broader repertoire of culture conditions to better capture native microbial richness and diversity measures.

The culture repository of individual bacterial isolates is enriched in the genera *Bacillus* and *Pantoea* (Table 1). These were also identified as core members of the citrus rhizosphere microbiome from citrus trees collected worldwide (21). The commonalities we found among the dominant genera in the culture-independent leaf bacteriome with other citrus leaf bacteriome studies include Methylobacterium and Hymenobacter spp. (55). *Pantoea*, *Bacillus*, and *Paenibacillus* were identified as dominant root-associated genera (21, 56, 57), and we also found these to be dominant genera in the leaf and stem tissue compartments in both our bulk culture-dependent and culture-independent works. To the best of our knowledge, only one other citrus microbiome study has reported on the mycobiome of citrus plants, where *Fusarium*, Exophiala, and *Colletotrichum* were dominant fungal genera in the rhizosphere of citrus collected globally (21). We also found these to be dominant fungal genera in our study. Several bacteria isolated from the rhizosphere of citrus in an HLB-impacted region in Florida were inhibitory against two bacteria, Agrobacterium tumefaciens and Sinorhizobium meliloti, which are phylogenetically related to “*Ca*. Liberibacter asiaticus” (58). The inhibitory isolates included those of Burkholderia metallica, Burkholderia territorii, Pseudomonas granadensis, Pseudomonas geniculata, Rhodococcus jialingiae, and Bacillus pumilus (58). We did not recover these bacteria in our culture collection, likely due to the different medium types and culture conditions utilized in the Riera et al. study (58), as well as potential differences in the microbiomes of different geographic regions and citrus cultivars with those in our study.

Many microbial natural products have been identified, purified, and developed into antimicrobials, with prototypical examples of naturally derived antibiotics being penicillin produced by Penicillium spp. and streptomycin produced by streptomycetes. Specific to the HLB pathosystem, the derived antimicrobial natural products streptomycin sulfate (FireWall 50WP; AgroSource, Inc.) and oxytetracycline hydrochloride (FireLine 17WP; AgroSource, Inc.) are being applied as spray applications to trees in Florida under Section 18 emergency registration in efforts to decrease pathogen titer and HLB severity. Microbial natural products can also serve as important starting points for bioactive drug discovery and synthesis pipelines.

The *L. crescens* agar diffusion assay provides an efficient platform to prescreen microbes, crude supernatant extracts, fractionated natural product extracts, and purified natural product compounds *in vitro* without the laborious and resource-intensive *in planta* or insect studies currently necessary for screening compounds against “*Ca*. Liberibacter asiaticus” growth. Although there are limitations inherent to using a surrogate bacterium, this work establishes a reservoir of candidate natural products and microbes for use in future *in vitro* pipelines once culture methodology is sufficiently refined and “*Ca*. Liberibacter asiaticus” sheds it “*Candidatus*” status to be designated Liberibacter asiaticus (36). To initiate our work on anti-*L. crescens* natural product purification from citrus-associated microbes, we focused our efforts on the *L. crescens*-inhibitory fungus *C. cladosporioides. Cladosporium* spp. are often identified as members of plant microbiomes and can promote plant health by directly antagonizing pathogens through the production of antimicrobial compounds or by producing plant growth-promoting compounds (59, 60).

Bioassay-guided fractionation of the crude extract of *C. cladosporioides* using the *L. crescens* inhibition assay yielded cladosporols A (compound 1), C (compound 2), and D (compound 3) as the major bioactive compounds. Compound 1 was originally isolated from *C. cladosporioides* and identified as a β-glucan biosynthesis inhibitor (61). Compounds 2 and 3 and two other cladosporols (including compound 1) were isolated from Cladosporium tenuissimum in an investigation of the biocontrol mechanisms of this hyperparasite of the rust fungus Uromyces appendiculatus (44). The stereochemical configurations of compound 2 and, by inference, compounds 1 and 3, were revised in 2017, and each was shown to have modest antibacterial activity against the bacteria Escherichia coli, Micrococcus luteus, Vibrio harveyi (62), and methicillin-resistant Staphylococcus aureus (MRSA) (45). Compound 1 has also attracted considerable interest as a peroxisome proliferator-activated receptor γ (PPARγ)-mediated inhibitor of cancer cell proliferation (see reference 63 and references therein). In this study, compounds 1 to 3 all displayed inhibitory activity against *L. crescens* in a dose-dependent fashion, with slightly higher inhibition by compound 2.

We also identified other *L. crescens*-inhibitory fungi and bacteria using our pipeline. Among the fungi screened, an isolate of *E. nigrum* secreted compounds that robustly inhibited *L. crescens*. The *Epicoccum* genus includes many known plant endophytes and has been noted for its profuse secondary metabolite repertoire (64). *E. nigrum* is also an effective biocontrol agent in several plant systems (65). Most notably, this fungus reduced symptom severity in periwinkle plants inoculated with the phloem-dwelling “*Candidatus* Phytoplasma mali,” indicating that it interacts directly or indirectly with the phloem and thus may have some promise in combating “*Ca*. Liberibacter asiaticus” in the phloem of citrus (66). Interestingly, the genus is abundant in the citrus packing house environment (64, 67). Among the bacteria screened from our cultured citrus microbiome, a *Pantoea* isolate with high taxonomic identity to Pantoea agglomerans and Pantoea vagans was found to secrete compounds inhibitory to *L. crescens* BT-1. Both of these *Pantoea* species are prevalent in cultivated crop systems (68–70) and have been used as biocontrol agents against plant diseases caused by bacteria, fungi, and oomycetes (68, 71). These *Pantoea* species have been developed into the commercial products, Bloomtime Biological FD biopesticide (Verdesian Life Sciences) and BlightBan C9-1 (Nufarm, Inc.), respectively. P. vagans suppresses fire blight of pear and apple as a standalone treatment (72). In contrast, in other studies, P. vagans was found to be ineffective at controlling fire blight in apple as a standalone treatment but efficacious when combined with streptomycin applications, reducing the number of streptomycin applications necessary to effectively suppress fire blight (73). Based on these results, our current and future research focuses include the isolation and identification of bioactive molecules produced by *E. nigrum*, *Pantoea* strains, and other microbes identified as inhibitory to *L. crescens* via our experimentation pipeline.

The elucidation of antipathogen chemistries produced by phytobiome constituents provides a foundation for future experiments aimed at enriching disease suppression in a diseased plant environment. In the HLB pathosystem, efforts to harness biologicals or their bioactive metabolites for the management of HLB via direct application face significant challenges (74). Among these is the fastidious nature of the pathogen “*Ca*. Liberibacter asiaticus,” as it is localized to the phloem, a difficult-to-access sector of the plant endosphere. Moreover, “*Ca*. Liberibacter asiaticus” is delivered directly to the phloem by its insect vector and has no known epiphytic phase. Thus, anti-“*Ca*. Liberibacter asiaticus” applications based on direct activity against the pathogen will require entry to the phloem. The next steps of this collective work are to evaluate the cladosporols (and/or other to-be-isolated natural products) for anti-“*Ca*. Liberibacter asiaticus” activity within citrus trees. Most importantly, assays designed to track the transit pathways of those molecules *in planta* are necessary to assess bioavailability across tissue compartments. It will also be pertinent to determine whether the “*Ca*. Liberibacter asiaticus”-inhibitory metabolites are produced by their respective microbes *in planta*. Empirical assessment of the potential for these microbes to be used, either directly as bioinoculants, or through cultural practices enriching their abundance *in planta*, to curtail “*Ca*. Liberibacter asiaticus,” and thus mitigate HLB, are the next steps for this research. Our overall goal is to determine how the citrus phytobiome interfaces with the “*Ca*. Liberibacter asiaticus” pathogen and eventually to understand the impact of microbial community composition on HLB outcomes. In the long term, these findings will lay the foundation for the development of sustainable plant disease mitigation strategies for commercial citriculture.

## MATERIALS AND METHODS

### Foliar, stem, and root sampling.

In March 2016, stems, roots, and leaves from 50 trees were collected from five different citrus orchards in Florida (locations are shown in Tables S1 and S3). Each tree was divided into four quadrants (north, south, east, and west), and two stems with attached leaves were collected from each of the quadrants and pooled and sealed in a plastic bag (total eight stems per tree). Feeder roots were sampled by removing topsoil from two sides of the tree approximately 30 to 50 cm away from the base of the trunk near the irrigation line. The feeder roots near this irrigation line were sampled, shaken to remove soil, and sealed in a plastic bag. Gloves were changed, and clippers and shovels were sterilized with 30% household bleach between each tree that was sampled. All samples were immediately placed on ice for transit to the laboratory, where they were stored at 4°C and processed within 24 h. DNA isolations were previously described by Ginnan et al. (20). Briefly, 100 mg (roots, leaves) or 200 mg (stems) (wet weight) of tissue was pulverized via bead beating and processed using the MagMAX-96 DNA multisample kit (Thermo Fisher Scientific), followed by DNA concentration assessment using the Infinite M1000 Pro plate reader (Tecan, Männedorf, Switzerland) and SpeedVac concentration for dry storage at –20°C prior to library construction.

### Microbial propagation for bulk culture collection.

Root samples were rinsed twice with sterile water to remove surface soil. Approximately 0.3 g of feeder roots was placed into a mesh grinding bag (Agdia, Inc., Elkhart, IN) with 2.0 ml of 1× phosphate-buffered saline (PBS). The tissue was ground with a hammer, and the resulting slurry was diluted 1:10 with 1× PBS. The leaves and stems (cut to 3-inch pieces) were processed in a similar manner but with 3 ml of 1× PBS. One hundred microliters of the 1:10-diluted slurry was spread plated on two solid medium types, tryptic soy agar (TSA) and potato dextrose agar with 0.1 g/liter tetracycline hydrochloride (PDA-tet). The plates were incubated at 28°C for 4 days. The consortia of microbes on each plate had 1 ml of 1× PBS added directly to the culture plate and were subsequently scraped with a cell scraper. The suspension was stored as glycerol stocks (25% final glycerol concentration) at –80°C. Simultaneously, 50 μl of this culture suspension in 1× PBS was used for DNA extraction using a Mo Bio DNeasy PowerSoil kit (Qiagen, Valencia, CA), according to the manufacturer’s recommended protocol.

### Microbial taxa identification in mixed microbial cultures and plant tissue samples.

**(i) HTS of the bacterial rRNA ITS region.** DNAs extracted from the bulk cultures and DNAs extracted from the cognate citrus tissue samples were used to construct Illumina bacterial rRNA ITS libraries as described by Ginnan et al. (20) and Ruegger et al. (75). The ITS region of the bacterial rRNA operon was utilized because it can provide higher taxonomic resolution than that in amplicon-based HTS studies of the 16S rRNA region (75).

**(ii) HTS of the fungal ITS region.** DNAs extracted from the bulk cultures and DNAs extracted from the cognate citrus tissue samples were used to construct Illumina fungal ITS libraries as described by Ginnan et al. (20).

### HTS data analyses.

Data processing for the bacterial data was performed with USEARCH v10.0 (76). We used the UPARSE pipeline for demultiplexing, length trimming, quality filtering, and operational taxonomic unit (OTU) picking using default parameters or recommended guidelines that were initially described in reference 77 and which have been updated at https://www.drive5.com/usearch/manual10/uparse_pipeline.html. Briefly, after demultiplexing and using the recommended 1.0 expected error threshold, sequences were trimmed to a uniform length of 145 bp and then dereplicated. Dereplicated sequences were subjected to error correction (denoised) and chimera filtering to generate zero-radius operational taxonomic units (ZOTUs) using UNOISE3 (78). An OTU table was then generated using the otutab command. ZOTUs having nonbacterial DNA were identified and enumerated by performing a local BLAST search (79) of their seed sequences against the nucleotide database. ZOTUs were removed if any of their highest-scoring BLAST hits contained taxonomic identifiers (IDs) within the citrus family, fungal kingdom, or PhiX. Taxonomic assignments to bacterial ZOTUs were made by finding the lowest common taxonomic level of the highest BLAST hits, excluding unclassified designations. Data were normalized by relative abundances within each sample by dividing the number of reads in each OTU by the total number of reads in that sample. The bacterial sequence mapping file with sample metadata and the OTU table can be found in Tables S1 and S2, respectively. Data processing for the fungal data was performed with USEARCH v10.0 (76). We used the UPARSE pipeline for demultiplexing, length trimming, quality filtering, and OTU picking using default parameters or recommended guidelines that were initially described in reference 77 and which have been updated at https://www.drive5.com/usearch/manual10/uparse_pipeline.html. Briefly, after demultiplexing and using the recommended 1.0 expected error threshold, sequences were trimmed to a uniform length of 249 bp and then dereplicated. Dereplicated sequences were subjected to error correction (denoised) and chimera filtering to generate ZOTUs using UNOISE3 (78). An OTU table was then generated using the otutab command. ZOTUs having nonfungal DNA were identified by performing a local BLAST search (79) of their seed sequences against the nucleotide database. ZOTUs were removed if any of their highest-scoring BLAST hits contained taxonomic IDs within the Viridiplantae kingdom or PhiX. Taxonomic assignments to fungal ZOTUs were made using the RDP Classifier version 2.12 (80) trained on the ver7_99_s_10.10.2017 release of the UNITE database (81). Data were normalized within each sample by dividing the number of reads in each OTU by the total number of reads in that sample. The fungal sequence mapping file with sample metadata and the OTU table can be found in Tables S4 and S5, respectively.

Taxonomy tables were generated using QIIME 1.9.1 (82) and analyzed using Prism (GraphPad, San Diego, CA). The bacterial and fungal taxon tables can be found in Tables S3 and S6, respectively. R was used for statistical analyses and data visualization, specifically, phyloseq (83). A Kruskal-Wallis *post hoc* Dunn test and Bonferroni correction were used to distinguish alpha-diversity differences (83). Percent values indicate the proportions of culture-independent OTUs found in cultured microbiome samples.

### Pure cultures of single isolates.

Isolates were initially recovered from bulk culture tubes on both tryptic soy agar (TSA) and potato dextrose agar (PDA) plates at 28°C for no longer than 5 days. For the bacteria, single colonies were streaked onto fresh plates of TSA and subcultured until pure, isolated, individual colonies were obtained. For storage of pure bacterial cultures, single colonies were grown overnight in Trypticase soy broth (TSB) at 28°C and shaken at 180 rpm. Cultures were then stored in 15% (final concentration) sterile glycerol at –80°C. For the fungi, plugs of agar were drawn from the margins of growing colonies and subcultured onto fresh PDA plates until single fungal isolates were recovered. Individual fungal isolates were stored in 3 different ways, as follows: (i) streaked onto PDA slants and grown at 28°C, (ii) grown at 28°C and harvested with sterile distilled water for water stocks stored at 4°C, and (iii) grown at 28°C, and then the plates were allowed to dry out for the preparation of dry flakes for storage at –80°C.

### Genus-level identification of pure culture isolates.

Genomic DNA of pure bacterial cultures was isolated by use of the DNeasy blood and tissue kit (Qiagen, Valencia, CA) or the Wizard genomic DNA purification kit (Promega Corporation, Madison, WI), and genomic DNA was isolated from the fungal cultures with the ZymoBIOMICS kit (Zymo Research, Tustin, CA) or the FastDNA Spin kit for soil (MP Biomedicals, LLC, Santa Ana, CA), both per the manufacturer’s instructions. Purified DNA was then sent for identification by Sanger sequencing using universal primers 8F and 1492R (40) at ID Genomics (Seattle, WA) or underwent PCR with either the 16S U1/U2 primers for bacteria (84, 85) or the ITS 1/ITS 4 primers for yeast and filamentous fungi (86) using PrimeSTAR GXL DNA polymerase (TaKaRa Bio USA, Inc., Mountain View, CA). The thermal cycling parameters were 98°C for 1 min, 30 cycles of 98°C for 10 s, either 60°C (for bacteria) or 55°C (for fungi) for 15 s, and 68°C for 2 min, followed by 68°C for 5 min. The resulting PCR products were purified with a DNA Clean & Concentrator-5 kit (Zymo Research, Irvine, CA) and submitted to the University of California, Riverside Institute for Integrative Genome Biology for Sanger sequencing with either 16S rRNA (bacteria) or ITS (fungi) primers. Genus-level identifications were determined using 97% similarity to the BLAST database or the Ribosomal Database Project (41) or the NCBI database (42).

### Species-level identification of *Cladosporium* sp.

To verify the species of the *Cladosporium*, primers were designed that were specific to *C. cladosporioides* using PRISE2, a program for designing species-specific PCR primers and probes (87) using seed sequences selected from OTUs generated in the culture-independent microbiome analysis from citrus. DNA was extracted from the isolates using the DNeasy PowerSoil kit (Qiagen, Valencia, CA), and PCR was performed using the following specific primers: forward (CladF3), 5′-CGGCTGGGTCTTCT-3′, and reverse (CladR3), 5′-CTTAAGTTCAGCGGGTAT-3′. The thermal cycling parameters were 94°C for 5 min, 40 cycles of 94°C for 20 s, 61.2°C for 20 s, and 72°C for 30 s, followed by 72°C for 10 min and 26°C for 20 min. Amplified regions were purified with a MinElute gel extraction kit (Qiagen, Valencia, CA), cloned into the pGEM-T plasmid for sequence analysis (Promega, Madison, WI), and then submitted for Sanger sequencing to the University of California, Riverside (UCR) Institute for Integrative Genome Biology.

### Liberibacter crescens inhibition bioassays.

Antagonism against *L. crescens* BT-1 (37) (kindly provided by E. Triplett) was assessed by an agar diffusion assay that tested spent-culture supernatants. Bacterial supernatant filtrates were taken from 3-day liquid cultures (propagated at 30°C, 180 rpm in bBM7 plus 1.0 methyl-β-cyclodextrin [mβc] liquid medium) and purified via solid-phase extraction (SPE; elution with methanol) (38). Fungal extracts were prepared from 3-week agar cultures, as follows: 1.56-cm^2^ sections of agar were extracted in 5 ml of methanol and shaken for 24 h at 180 rpm at room temperature. Fifty microliters of either fungal extracts or bacterial supernatant filtrates was applied to sterile paper disks (Becton, Dickinson, Franklin Lakes, NJ) and allowed to dry in a biosafety cabinet. bBM7 plus 1.0 mβc top agar (0.8% agar) was prepared, cooled to 60°C, and amended with a 4-day *L. crescens* liquid culture (bBM7 plus 1.0 mβc, 28°C, 180 rpm shaking) at 10% of the top agar volume. This amended top agar was then dispensed to evenly coat previously poured bBM7 plus 1.0 mβc agar plates, after which supernatant-loaded filter disks were placed. Cultures were incubated for 6 days at 28°C to allow for the development of clear zones of inhibition, after which zone diameters were recorded. Isolates were tested in three independent experiments with three technical replicates for each isolate for each experiment.

### Natural product fractionation and characterization.

Agar plugs (0.5 cm^2^) of *C. cladosporioides* isolate CF0052 were used to inoculate liquid cultures (12 × 250 ml PDB in 500-ml Erlenmeyer flasks). Cultures were incubated for 32 days at 20°C with shaking at 180 rpm and extracted exhaustively with ethyl acetate (EtOAc) (3 × 250 ml), and the resulting combined extracts were evaporated *in vacuo* to yield a dark-brown residue. The crude extract was fractionated by flash silica-gel column chromatography (CombiFlashRf200; Teledyne Instruments, Inc.) at a flow rate of 30 ml/min with gradient elution (0% to 100% EtOAc-hexanes over 20 min, followed by 0% to 20% methanol-dichloromethane [MeOH-DCM] over 9 min) to give 6 fractions. Fractions 3 (44.3 mg), 4 (10.6 mg), and 5 (25.4 mg) were subjected to HPLC (Prominence-i LC-2030C liquid chromatograph equipped with a diode-array detector; Shimadzu Scientific Instruments) to give compounds 1 (1.4 mg), 2 (3.2 mg), and 3 (1.0 mg). Liquid chromatography-electrospray ionization-time of flight-mass spectrometry (LC-ESI-TOF-MS) was performed using an Agilent 1260 Infinity liquid chromatograph coupled to a 6530 quadrupole-TOF (Q-TOF) mass spectrometer. NMR spectra were obtained using a JEOL ECS spectrometer (400 MHz for ^1^H and 100 MHz for ^13^C), using CDCl_3_ from Cambridge Isotope Laboratories, Inc., and referenced to trimethylsilyl (TMS). NMR summary tables and complete LC-MS and NMR data for compounds 1 to 3 can be found in the supplemental material.

### Data availability.

The bacterial and fungal HTS data sets have been deposited in the National Center for Biotechnology Information (NCBI)’s Sequence Read Archive (SRA) under BioProject no. PRJNA546069.

## Supplementary Material

Supplemental file 1

Supplemental file 2

Supplemental file 3

Supplemental files 4

Supplemental file 5

Supplemental file 6

Supplemental file 7
