# Supplementary material for: An In Vitro Pipeline for Screening and Selection of Citrus-Associated Microbiota with Potential Anti-“Candidatus Liberibacter asiaticus” Properties
Source: Appl Environ Microbiol. 2020 Apr 1;86(8):e02883-19. doi: 10.1128/AEM.02883-19 (PMC7117939; doi:10.1128/AEM.02883-19)
Supplement: Supplemental file 7 [file AEM.02883-19-s0007.pdf]

Supplemental Information

- ^{13}C NMR comparison table of observed and literature values for cladosporols A (**1**) and C (**2**) ($CDCl_3$)
- ^{1}H NMR comparison table of observed and literature values for cladosporol D (**3**) (acetone-d_6)
- ^{1}H NMR spectrum of cladosporol A (**1**) at 400 MHz ($CDCl_3$)
- ^{13}C NMR spectrum of cladosporol A (**1**) at 100 MHz ($CDCl_3$)
- ESI-TOFMS of cladosporol A (**1**)
- ^{1}H NMR spectrum of cladosporol C (**2**) at 400 MHz ($CDCl_3$)
- ^{13}C NMR spectrum of cladosporol C (**2**) at 100 MHz ($CDCl_3$)
- ESI-TOFMS of cladosporol C (**2**)
- ^{1}H NMR spectrum of cladosporol D (**3**) at 400 MHz (acetone-d_6)
- ^{1}H-^{1}H COSY NMR spectrum of cladosporol D (**3**) at 400 MHz (acetone-d_6)
- ESI-TOFMS of cladosporol D (**3**)

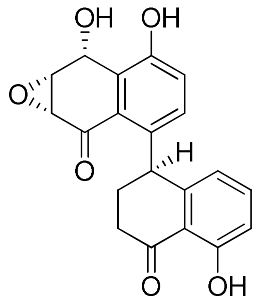

cladosporol A (**1**)

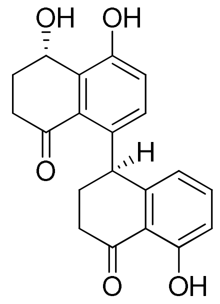

cladosporol C (**2**)

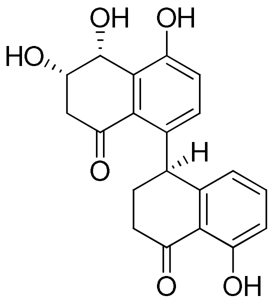

cladosporol D (**3**)

Table S8. ^{13}C NMR spectroscopic data for **1** and **2** in $CDCl_3$. Observed values are provided along with published values in ppm (^{a}(1), ^{b}(2))

Position	**1** *obs.*	**1** *lit.*^{a}	**2** *obs.*	**2** *lit.*^{b}
1	205.4	205.3	199.4	199.7
2	56.3	56.0	37.8	37.5
3	55.4	55.2	31.9	31.4
4	67.7	67.5	68.1	67.4
4a	122.8	122.6	129.5	129.8
5	155.7	155.5	154.6	154.4
6	122.7	122.6	121.7	121.3
7	132.4	132.2	131.8	131.5
8	137.4	137.2	137.5	137.1
8a	128.4	128.2	130.8	130.6
1’	195.1	195.0	205.6	205.6
2’	37.0	36.8	36.8	36.6
3’	31.2	31.1	30.6	29.7
4’	40.2	40.1	40.0	40.0
4’a	148.4	148.3	148.6	148.5
5’	120.1	120.0	120.1	120.0
6’	136.6	136.4	136.6	136.5
7’	115.9	115.8	115.9	115.7
8’	162.9	162.7	162.9	162.7
8’a	117.8	117.6	117.9	117.7

Table S9. ^{1}H NMR spectroscopic data for **1** and **2** in $CDCl_3$, and **3** in acetone-d_6. Observed values are provided along with published values in ppm, [a](1), [b])(3)), [c]*J* values not specified in Sakagami et al. [d]Not observed. [e]Exact chemical shift and splitting obscured due to peak overlap. [f]Multiplicity not specified in Nasini et al, only selected *J*-values.

	**1** *obs.*		**1** *lit.*[a]		**2** *obs.*		**2** *lit.*[b]		**3** *obs.*		**3** *lit.*[b]	
Position	δ ^{1}H	*J* (Hz)	δ ^{1}H	*Mult*[c]	δ ^{1}H	*J* (Hz)	δ ^{1}H	*J* (Hz)[f]	δ ^{1}H	*J* (Hz)	δ ^{1}H	*J* (Hz)[f]
2a	3.80	*d* (4.5)	3.85	*d*	2.84	*ddd* (15.7, 6.6, 4.3)	2.91	(15.5, 7.2, 4.2)	2.83	*d* (8.5)	3.00	(15.6, 8.3)
2b					2.6[e]	[e]	2.64	(15.5)			2.86	(15.6, 4.2)
3a	4.00	*dd* (4.5, 2.2)	4.01	*dd*	2.48	*m*	2.48		4.18	*m*	4.31	(8.3, 4.2, 3.2)
3b					2.22	*m*	2.25		-		-	
4	5.36	*br s*	5.40	*d*	5.28	*m*	5.33	(8.2, 5.0)	5.24	*dd* (5.4, 3.6)	5.39	(3.2)
6	6.97	*d* (8.8)	7.00	*dd*	6.91	*d* (8.6)	6.95	(8.5)	6.88	*d* (8.7)	7.02	(8.5)
7	6.87	*d* (8.8)	6.92		6.78	*d* (8.6)	6.80	(8.5)	6.74	*d* (8.7)	6.86	(8.5)
2’a	2.70	*m*	2.74	*m*	2.6[e]	[e]	2.68	(17.5, 6.8, 6.2)	2.51	*t* (6.0	2.66	(17.5, 8.0, 6.0)
2’b					2.6[e]	[e]	2.62	(17.5, 6.8, 6.2)			2.61	(17.5, 6.2, 5.8)
3’a	2.15	*m*	2.19	*m*	2.32	*m*	2.34	(13.5, 6.2, 5.0)	2.15	*t* (6.0)	2.26	(13.5, 6.2, 6.0, 5.5)
3’b	2.45	*m*	2.49	*m*	2.07	*m*	2.13	(13.5, 6.8, 7.2)			2.20	(13.5, 8.0, 5.8)
4’	4.81	*dd* (8.0, 4.8)	4.85	*dd*	5.45	*m*	5.46	(7.2, 5.0)	5.47	*m*	5.58	(8.0, 5.5)
5’	6.16	*d* (7.8)	6.19	*d*	6.28	*d* (7.8)	6.34	(7.7, 1.2)	6.23	*d* (7.9)	6.35	(7.7, 1.2)
6’	7.22	*d* (8.5)	7.24	*dd*	7.23	*t* (~8.0)	7.28	(8.2, 7.7)	7.23	*t* (7.9)	7.34	(8.2, 7.7)
7’	6.74	*d* (8.5)	6.74	*d*	6.75	*d* (8.3)	6.79	(8.2, 1.2)	6.63	*d* (8.2)	6.76	(8.2, 1.2)
3-OH	-		-				-		[d]		3.20	
4-OH	[d]		3.39	*d*	3.96	*br s*	3.95		4.85	*d* (5.3)	4.52	
5-OH	8.64	*s*	8.69	*s*	7.96	*s*	8.70		9.09	*s*	9.20	
8’-OH	12.54	*s*	12.57	*s*	12.52	*s*	12.58		12.49	*s*	12.62	

^{1}H NMR of cladosporol A (**1**) at 400 MHz ($CDCl_3$)

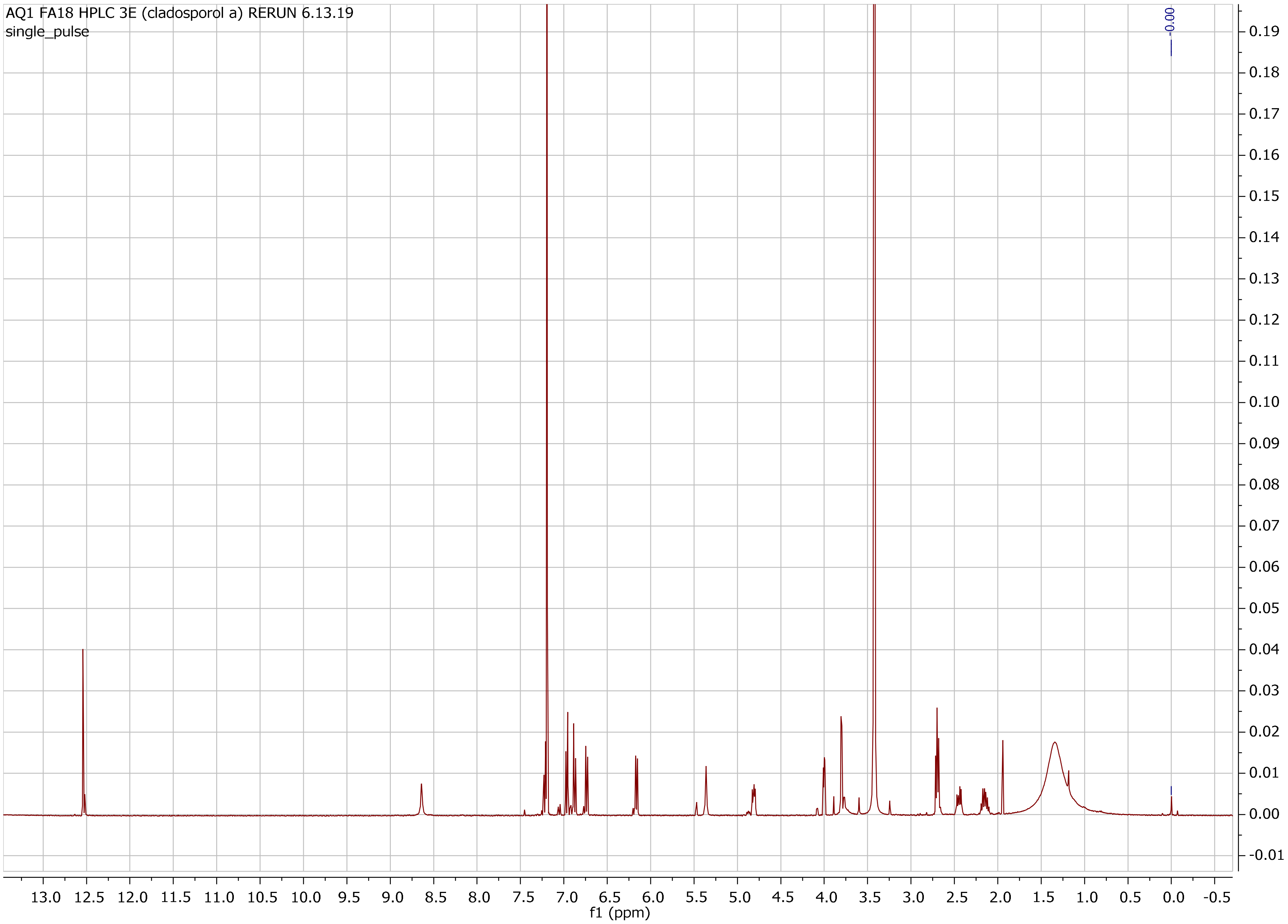

^{13}C NMR of cladosporol A (**1**) at 100 MHz ($CDCl_3$)

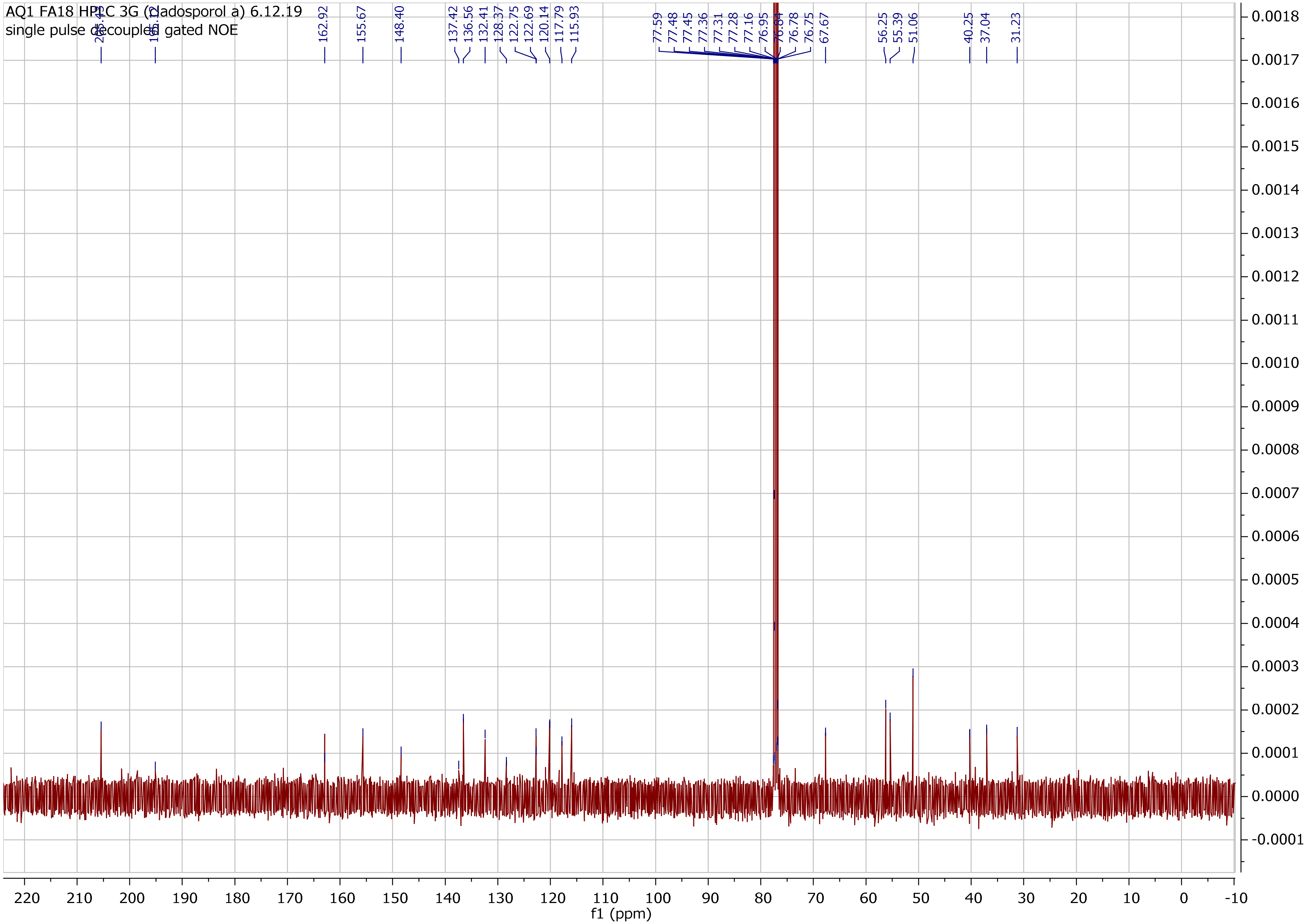

ESI-TOFMS of cladosporol A (**1**)

[M-H]$^-$ *m/z* 351.05 (calcd. for $C_{20}H_{15}O_6$ 351.09). Also see dimer [M_2-H]$^-$ at *m/z* 703.04 (calcd. for $C_{40}H_{31}O_{12}$ 703.18)

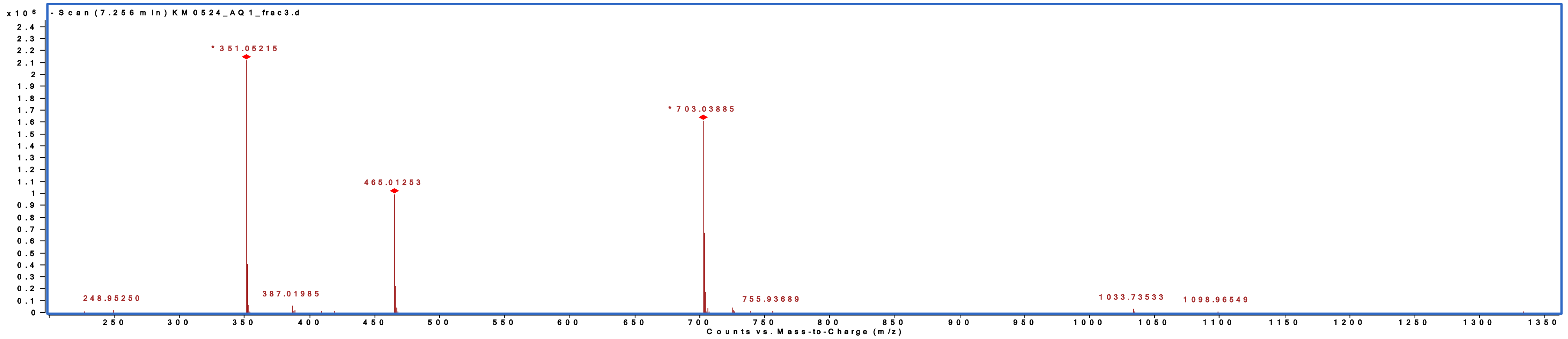

^{1}H NMR spectrum of cladosporol C (**2**) at 400 MHz ($CDCl_3$)

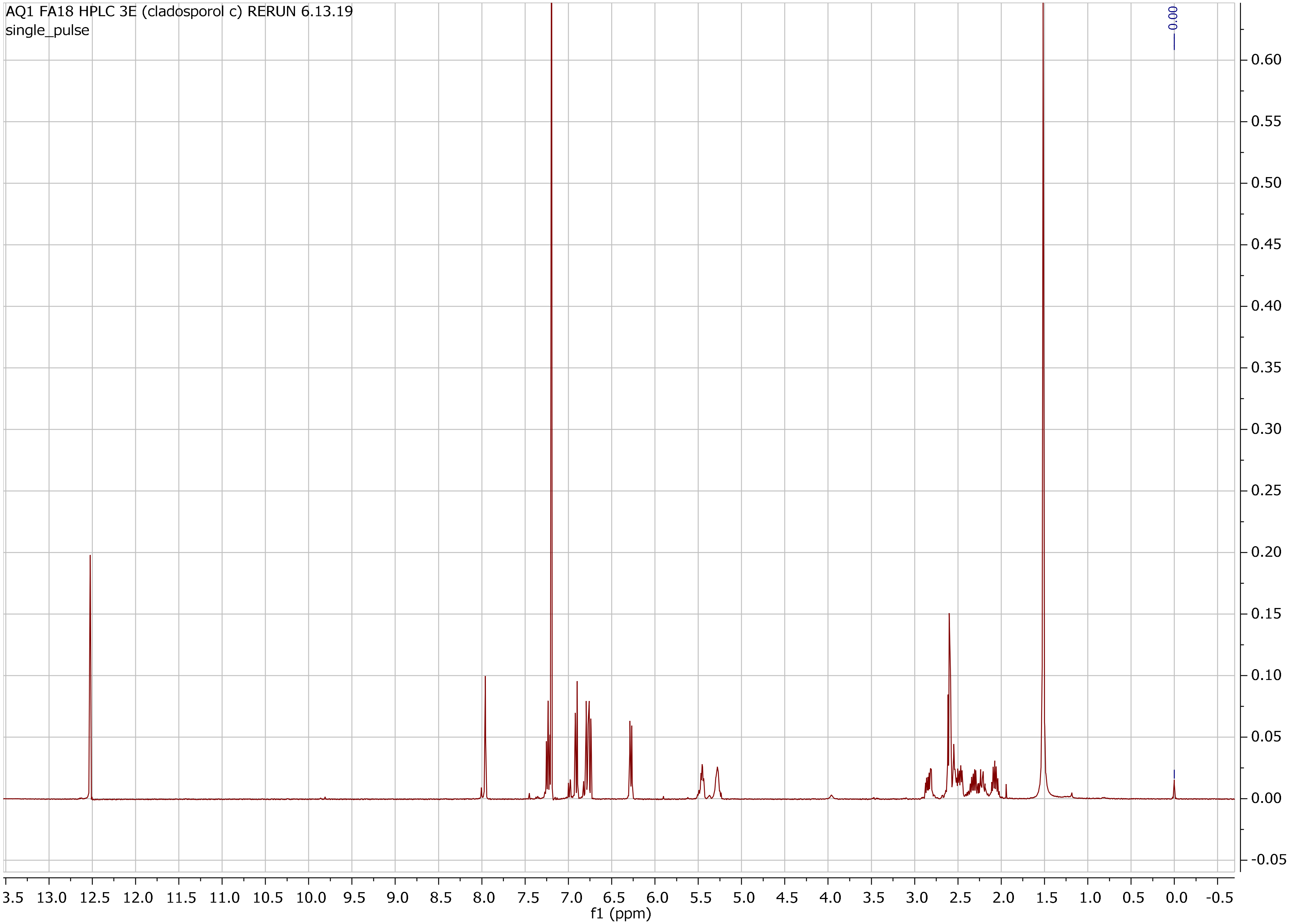

^{13}C NMR spectrum of cladosporol C (**2**) at 100 MHz ($CDCl_3$)

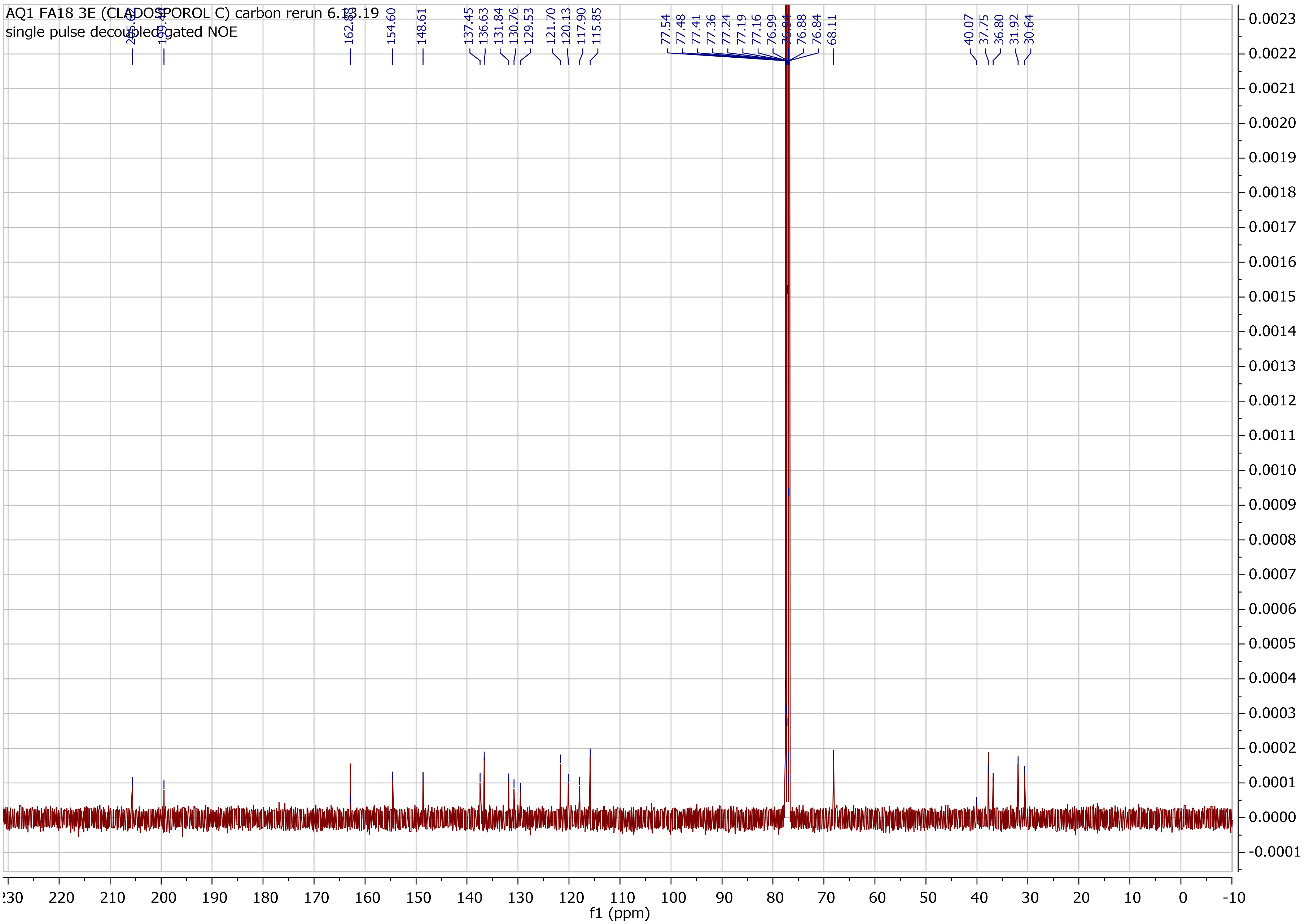

ESI-TOFMS of cladosporol C (**2**)

[M-H]$^{-}$ *m/z* 337.08 (calcd. for $C_{20}H_{17}O_5$ 337.11). Also see dimer [M$_2$-H]$^{-}$ at *m/z* 675.09 (calcd. for $C_{40}H_{35}O_{10}$ 675.22)

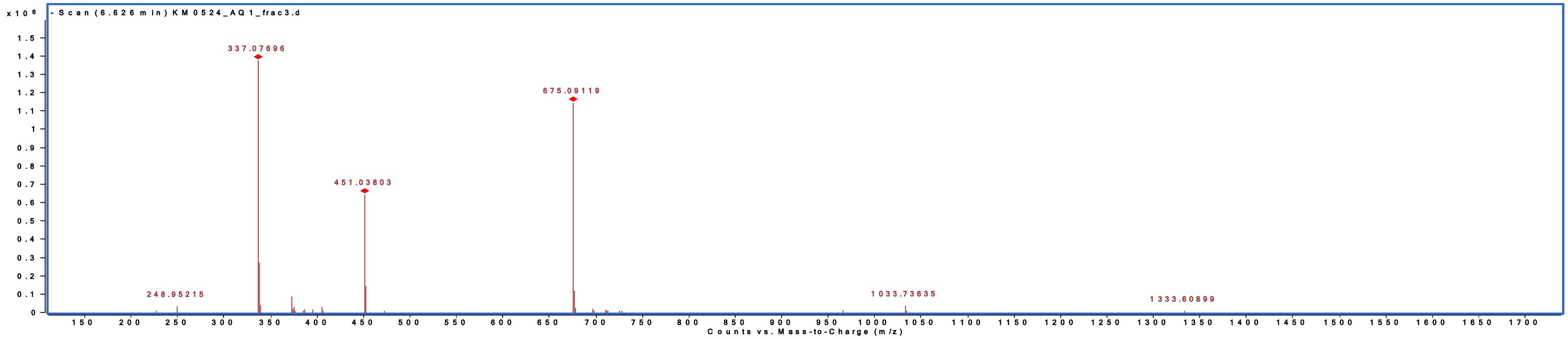

^{1}H NMR spectrum of cladosporol D (**3**) at 400 MHz (acetone-d_6)

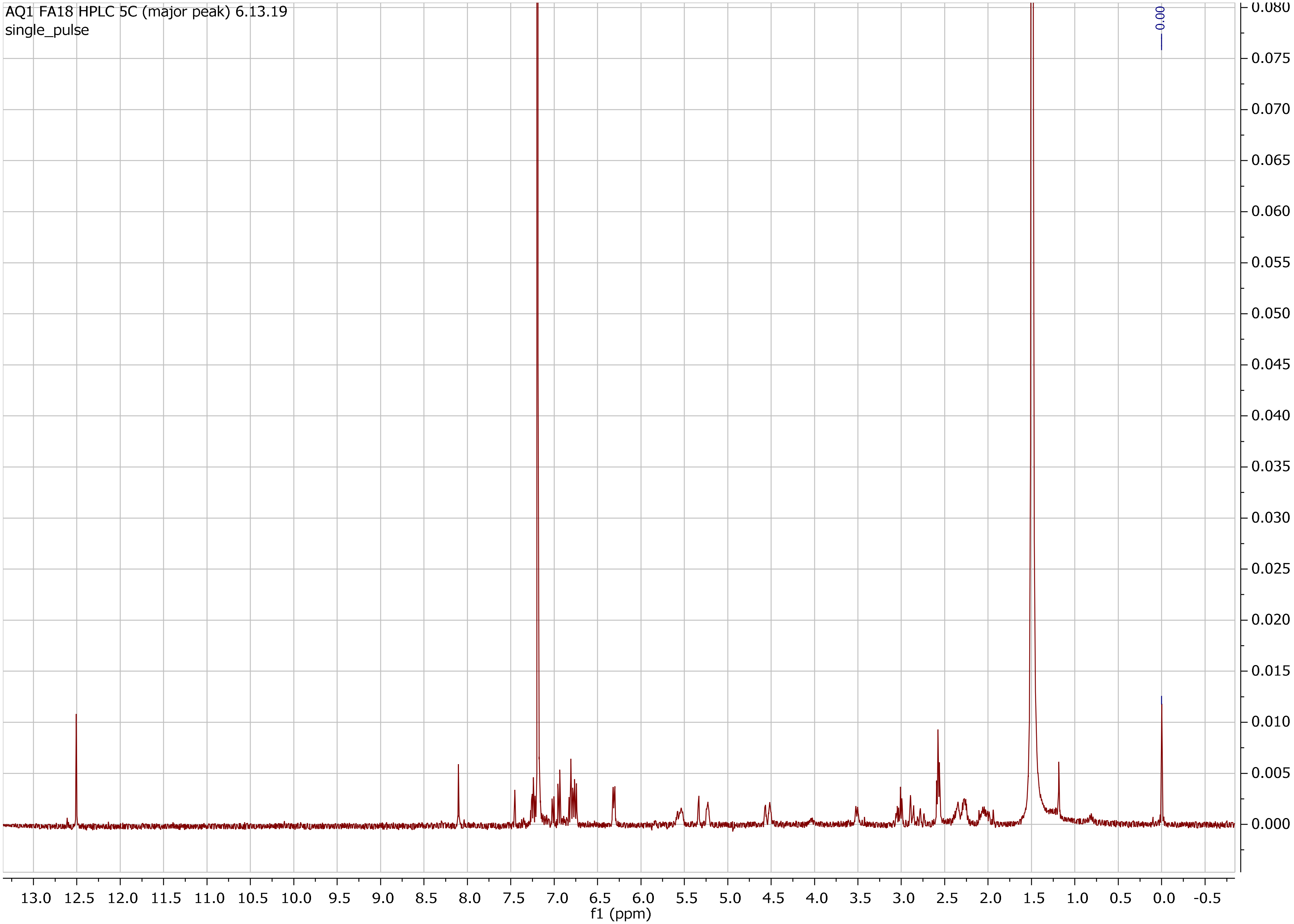

^{1}H-^{1}H COSY NMR spectrum of cladosporol D (**3**) at 400 MHz (acetone-d_6)

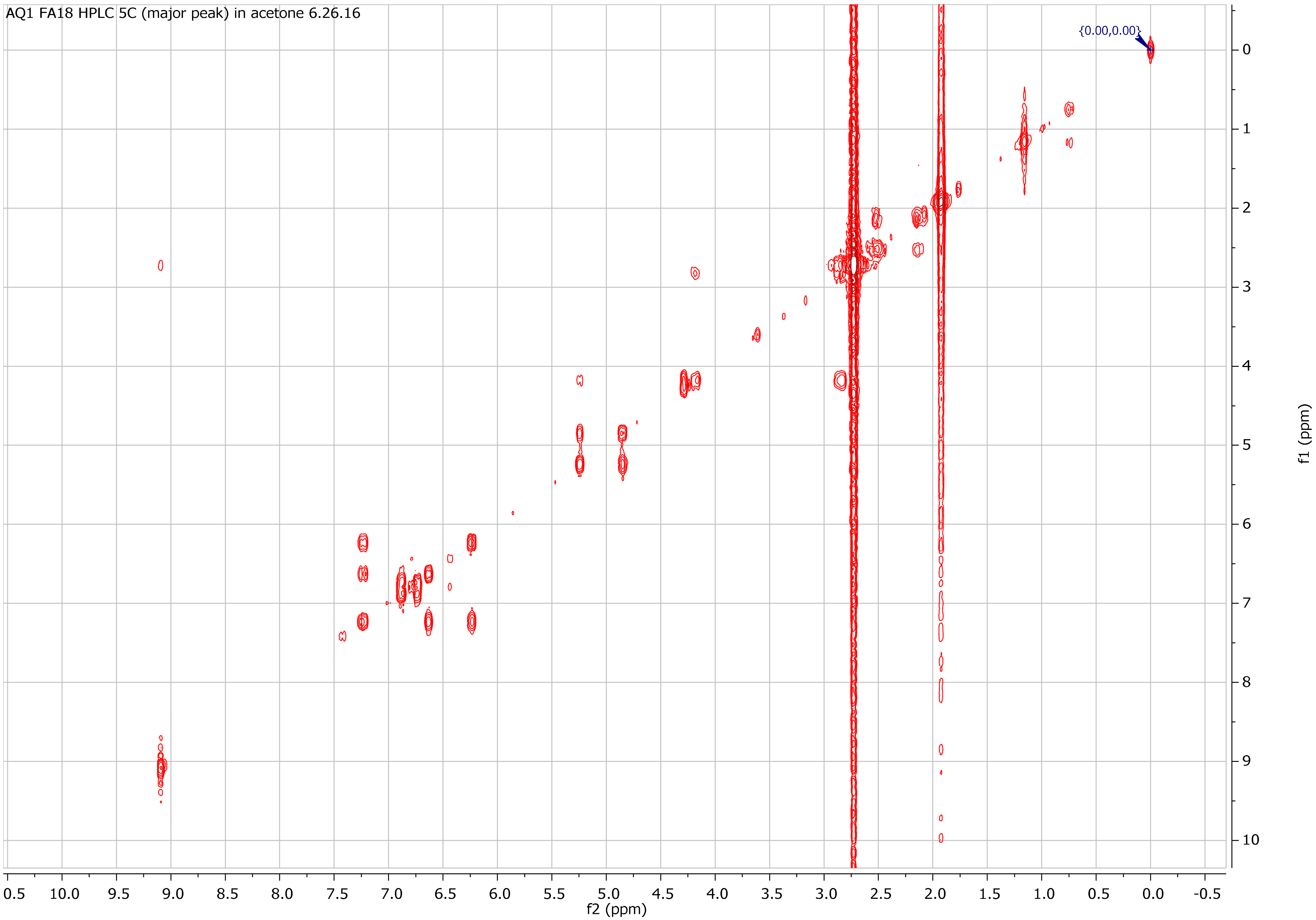

ESI-TOFMS of cladosporol D (**3**)

[M-H]$^-$ *m/z* 353.07 (calcd. for $C_{20}H_{17}O_6$ 353.10). Also see dimer $[M_2\text{-}H]^-$ at *m/z* 707.08 (calcd. for $C_{40}H_{35}O_6$ 707.21)

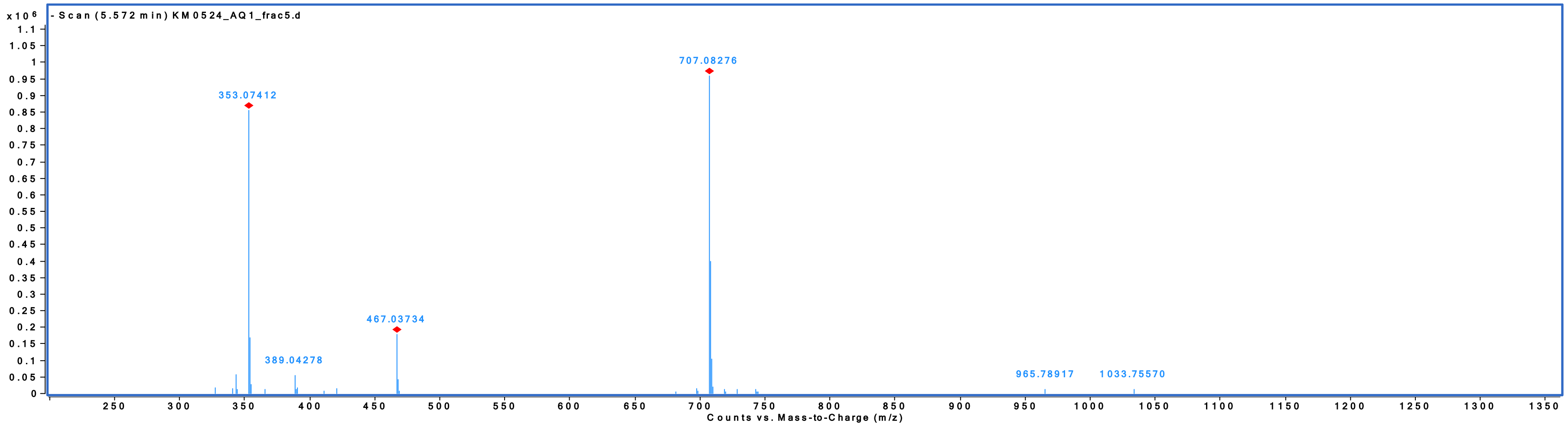

1. Sakagami Y, Sano A, Hara O, Mikawa T, Marumo S. 1995. Cladosporol, β-1, 3-glucan biosynthesis inhibitor, isolated from fungus, Cladosporium cladosporioides. Tetrahedron Lett 36:1469–1472.

2. Li H-L, Li X-M, Mándi A, Antus S, Li X, Zhang P, Liu Y, Kurtán T, Wang B-G. 2017. Characterization of Cladosporols from the Marine Algal-Derived Endophytic Fungus Cladosporium cladosporioides EN-399 and Configurational Revision of the Previously Reported Cladosporol Derivatives. J Org Chem 82:9946–9954.

3. Nasini G, Arnone A, Assante G, Bava A, Moricca S, Ragazzi A. 2004. Secondary mould metabolites of Cladosporium tenuissimum, a hyperparasite of rust fungi. Phytochemistry 65:2107–2111.
